# Neuroprotective effects of methylene blue against cadmium‐induced Alzheimer's disease‐like symptoms in rats

**DOI:** 10.1002/alz70855_105439

**Published:** 2025-12-24

**Authors:** Soumia Ed‐Day, Fatima Ezzahra Kacimi, Radia Elgui, Latifa Didou, Samira Boulbaroud, Fatima‐Zahra Azzaoui

**Affiliations:** ^1^ Unit of Neuroscience, Neuroimmunology & Behaviour, Biology & Health Laboratory, Department of Biology, Faculty of Science, Ibn Tofail University, Kenitra, Rabat/Sale/Kenitra, Morocco; ^2^ Department of Biology, Polydisciplinary Faculty, Sultan Moulay Slimane University, Beni Mellal, Beni Mellal/ Khenifra, Morocco

## Abstract

**Background:**

The environment and human health are closely related, yet this balance is increasingly threatened by invisible pollutants such as heavy metals. Cadmium is a particularly insidious neurotoxic agent; it contaminates soil, water, and air while silently threatening ecosystems and human health. Recent studies highlight a potential link between cadmium exposure and Alzheimer's disease (AD). Methylene blue (Mb), a therapeutic agent, is well regarded for its beneficial effects on cognitive disorders. This study aimed to evaluate the neuroprotective potential of Mb against cadmium chloride (CdCl_2_)‐induced AD‐like symptoms in rats. Specifically, it investigates the anti‐amnesic, antioxidant, and cholinergic regulatory properties of Mb.

**Method:**

33 adult Wistar rats were divided into three groups (*n* =  11). The control group received an intracerebroventricular (ICV) injection of sterile saline (5 µl/ventricle). The disease group was administered CdCl_2_ (10 µg/kg) via ICV administration. The treated group received an ICV‐CdCl_2_ injection and a daily oral dose of Mb (2 mg/kg) for two months. At the end of the experiment, all rats were assessed for memory deficits, histopathological, and neurochemical changes.

**Result:**

Our results demonstrated that CdCl_2_ induces hallmark features of AD, such as memory deficits, cholinergic insufficiency, and oxidative stress. On the other hand, Mb significantly enhanced memory, as evidenced by an increase in spontaneous alternation (*p* < 0.05), the recognition index for short and long‐term memory (*p* < 0.001), and the time spent in the correct quadrant (*p* < 0.05) compared to the disease group, as measured by the Y‐maze, novel object recognition, and Morris water maze tests. Furthermore, Mb significantly elevated acetylcholine levels (*p* < 0.01) in the brain. It also led to a significant decrease in malondialdehyde and nitric oxide levels (*p* < 0.01), along with an increase in superoxide dismutase (*p* < 0.01). Histological examination of the hippocampus sub‐regions following Mb treatment revealed normal structure and arrangement of neurons. Additionally, Mb reduced cell loss compared to the disease group.

**Conclusion:**

The administration of CdCl_2_ directly into the brain via the intracerebroventricular route induced features of AD, while Methylene Blue demonstrated neuroprotective effects by enhancing memory, regulating acetylcholine levels, and mitigating oxidative stress.

